# A novel human laboratory model for screening medications for alcohol use disorder

**DOI:** 10.1186/s13063-020-04842-w

**Published:** 2020-11-23

**Authors:** Diana Ho, Brandon Towns, Erica N. Grodin, Lara A. Ray

**Affiliations:** 1 Department of Psychology, University of California, Los Angeles, 1285 Franz Hall, Box 951563, Los Angeles, CA 90095-1563 USA; 2 Department of Psychiatry and Biobehavioral Sciences, University of California, Los Angeles, Los Angeles, CA USA

**Keywords:** Alcohol use disorder, Medications development, Medications screening, Naltrexone, Varenicline, Clinical trial

## Abstract

**Background:**

Alcohol use disorder (AUD) is a highly prevalent, chronic relapsing disorder with a high disease burden in the USA. Pharmacotherapy is a promising treatment method for AUD; however, the few FDA-approved medications are only modestly effective. Medications development for AUD is a high priority research area, but the cumbersome drug development process hinders many potential compounds from reaching approval. One area with major opportunities for improvement is the process of screening novel compounds for initial efficacy, also known as early phase 2 trials. Early phase 2 trials incorporate human laboratory paradigms to assess relevant clinical constructs, such as craving and subjective responses to alcohol. However, these controlled paradigms often lack the ecological validity of clinical trials. Therefore, early phase 2 trials can be more efficient and clinically meaningful if they combine the internal validity of experimental laboratory testing with the external validity of clinical trials. To that end, the current study aims to develop and validate a novel early efficacy paradigm, informed by smoking cessation literature, to screen novel medications for AUD. As an established AUD medication, naltrexone will serve as an active control to test both the practice quit attempt model and the efficacy of a promising AUD pharmacotherapy, varenicline.

**Methods:**

Individuals with current AUD reporting intrinsic motivation to change their drinking will complete a week-long “practice quit attempt” while on study medication. Participants are randomized and blinded to either naltrexone, varenicline, or placebo. During the practice quit attempt, participants will complete daily visits over the phone and fill out online questionnaires regarding their drinking, alcohol craving, and mood. Additionally, participants will undergo two alcohol cue-reactivity sessions.

**Discussion:**

The successful completion of this study will advance medications development by proposing and validating a novel early efficacy model for screening AUD pharmacotherapies, which in turn can serve as an efficient strategy for making go/no-go decisions as to whether to proceed with clinical trials.

**Trial registration:**

ClinicalTrials.gov NCT04249882. Registered on 31 January 2020.

## Administrative information

The order of the items has been modified to group similar items (see http://www.equator-network.org/reporting-guidelines/spirit-2013-statement-defining-standard-protocol-items-for-clinical-trials/).

**Table:** 

Title {1}	A Novel Human Laboratory Model for Screening Medications for Alcohol Use Disorder
Trial registration {2a and 2b}.	ClinicalTrials.gov: NCT04249882
Protocol version {3}	Version 5; dated 05/2020
Funding {4}	The National Institute on Alcohol Abuse and Alcoholism of the National Institutes of Health, Award Number R21AA027180
Author details {5a}	^1^Department of Psychology, University of California, Los Angeles, Los Angeles, California ^2^Department of Psychiatry and Biobehavioral Sciences, University of California, Los Angeles, Los Angeles, CA
Name and contact information for the trial sponsor {5b}	Dr. Lara Ray, Principal Investigator, lararay@psych.ucla.edu
Role of sponsor {5c}	The sponsor is responsible for the study design; and will play a part in the collection, management, analysis, and interpretation of data; writing of the report; and the decision to submit the report for publication.

## Introduction

### Background and rationale {6a}

Alcohol use disorder (AUD) is a highly prevalent, chronic relapsing disorder with a high disease burden in the United States [1, 2]. Despite current and lifetime prevalence rates of 13.9% and 29.1%, respectively, it remains largely untreated as only 7.7% of those with 12-month and 19.8% of those with lifetime diagnoses sought treatment in 2012–2013 [1]. In spite of low treatment rates, pharmacotherapy offers a promising treatment method for AUD [3, 4]. The Federal Drug Administration (FDA) has approved of four medications for AUD: disulfiram (Antabuse®), oral naltrexone (ReVia®), extended-release injectable naltrexone (Vivitrol®), and acamprosate (Campral®) [5]. However, these currently approved pharmacotherapies are only modestly effective, so there is still a great need to develop more effective interventions [6]. Medications development is a very costly, cumbersome, and inefficient process that can take nearly 20 years from discovery to market [3, 7]. In particular, the development of treatments for alcoholism has been difficult with over 20 medications having been tested in humans yet only three were able to receive FDA approval, the last of which was granted over a decade ago [8]. Therefore, there is a pressing need to develop valid and efficient methods to decrease the cost and length of medications development to better shepherd novel compounds from the lab to dissemination.

The development of novel medications for AUD is a high priority research area, but the drug development process is long and challenging, with many compounds stuck in the transition from preclinical to clinical testing, also known as the “valley of death” [3]. Beyond the “valley of death,” there is an overall need to develop effective methodologies for efficiently running clinical trials, particularly in screening novel compounds in early phase 2 trials [3, 7, 9]. Early phase 2 trials, also known as “proof-of-concept” studies, help determine if a novel medication is safe, tolerable, and efficacious using clinically relevant phenotypes such as cue-induced craving or subjective response to alcohol [7, 10]. These trials largely incorporate human laboratory paradigms to assess medication efficacy, providing valuable information on whether or not the medication warrants a larger clinical trial [7]. However, human laboratory paradigms have not always demonstrated translational validity and often lack the ecological validity of clinical trials where medication efficacy is established through clinically meaningful endpoints [3, 8, 10]. Therefore, there are major opportunities to refine this process of screening novel medications by combining the internal validity of human laboratory models and the external validity of clinical trials. To that end, the current study aims to develop and validate a novel early efficacy paradigm to screen medications for AUD.

This early efficacy paradigm is the practice quit attempt model adapted from the smoking cessation medication development literature [11–13]. In the original practice quit attempt model, individuals who report intrinsic motivation to quit smoking undergo a 7-day practice quit attempt while taking study medication [11–13]. Individuals with high intrinsic motivation to quit smoking fared better on active medication, compared to placebo, on increased abstinence, while individuals with low intrinsic motivation showed no effect of active medication [12]. Additionally, the practice quit model demonstrated specificity in which bupropion, an FDA-approved medication for smoking cessation, increased number of days abstinent, whereas modafinil, a medication ineffective for smoking cessation, was no different than placebo [13]. The success of the practice quit attempt model for screening medications for nicotine dependence provides a basis for the development of a similar approach modified for AUD.

In addition to the standard procedures of the practice quit attempt, we have included an established human laboratory paradigm to ensure that the novel model will be sensitive to medication effects. The cue-reactivity (CR) paradigm measures alcohol craving by having individuals hold and smell their preferred alcoholic beverage and a control beverage (water) [14]. Naltrexone (NTX), which is FDA-approved for AUD, is effective at significantly reducing alcohol-cue elicited craving compared to matched placebo [15]. Similar evidence exists for varenicline, a promising pharmacotherapy for AUD [16]. Thus, our current study will include CR in order to detect medication effects on cue-induced craving which will also verify the sensitivity of the novel practice quit attempt model to those medication effects.

In order to appropriately test and validate this model for AUD, we will use an established, FDA-approved medication. NTX is an opioid antagonist with high affinity for mu-opioid and kappa-opioid receptors [17]. Preclinical studies have shown that opioid antagonists at the mu-opioid receptor reduce ethanol consumption [18]. In humans, alcohol consumption increases the release of endogenous opioids in the mesolimbic dopamine reward system which contributes to the subjective pleasurable effects of alcohol [19]. Therefore, NTX’s therapeutic benefit as an opioid antagonist is proposed to block these rewarding effects and reduce alcohol consumption. Previous studies of NTX have shown that it reduces drinks per drinking day, alcohol craving, rates of relapse, and the subjective pleasurable effects of alcohol [20–26]. The effects of NTX appear to be moderated by craving such that higher levels of craving were found to be associated with greater reduction in alcohol consumption [22]. As an established medication for AUD, NTX is an ideal candidate to test the novel practice quit attempt model.

To further validate this novel early efficacy model, we will also test a promising medication to treat AUD. Varenicline (VAR) is a partial agonist at α4β2 and a full agonist at α7 nicotinic acetylcholine receptors, which is FDA-approved for smoking cessation [27]. In preclinical studies, activation of nicotinic acetylcholine receptors reduced ethanol consumption [28–30]. In human laboratory studies, VAR reduced alcohol self-administration and craving, compared to placebo [31]. In smoking cessation trials, it also reduced alcohol consumption and craving [32, 33]. Additionally, a multi-site randomized controlled trial (RCT) of VAR in individuals with AUD found that it reduced drinks per drinking day, alcohol craving, and percentage of heavy drinking days [34]. Together, these studies suggest that VAR is a promising pharmacotherapy for the treatment of AUD. Therefore, including varenicline, a widely studied and promising AUD pharmacotherapy, as a third arm in this study will enable us to further validate this novel alcohol quit paradigm.

In designing the current study as a 3-arm trial, we benefit not only from establishing the efficacy of NTX and VAR against placebo, but also from a head-to-head comparison of NTX and VAR in a cost-effective manner. The 3-arm trial design has been selected to overcome weaknesses present in noninferiority trials (also known as equivalence or active control trials) where a novel drug is compared to an active control that is the current standard treatment [35–37]. In active control trials, medication efficacy of the novel drug is determined by demonstrating noninferiority to the active control, which rests on the critical assumption that the active control has an actual drug effect [35]. However, as there is no placebo control, this assumption cannot be proven; therefore, noninferiority/equivalence trials lack assay sensitivity, or the ability to distinguish between effective and ineffective treatments [35, 36, 38]. The 3-arm design essentially combines the advantages of placebo and active controlled trials [37]. The placebo arm will allow us to showcase if VAR is an effective or ineffective medication in the context of a good internal standard (i.e., NTX is superior to placebo) [36]. Additionally, if neither NTX nor VAR are shown to be superior to placebo, then we can conclude that the practice quit paradigm is not a valid method for screening medications for AUD [36].

### Objectives {7}

The three primary objectives are:

1. To test whether NTX (50 mg) or VAR (2 mg) results in a (a) higher percentage days abstinent (PDA) and (b) lower number of drinks per drinking day (DPDD) during the 7-day practice quit attempt, as compared to placebo.
2. To test whether NTX (50 mg) or VAR (2 mg) reduces cue-induced craving for alcohol, as compared to placebo, and to confirm NTX effects using an established paradigm (i.e., alcohol CR paradigm).
3. To test the association between medication effects on CR in the laboratory and drinking behavior during the practice quit attempt.

The secondary objective is to directly compare NTX and VAR on PDA and number of DPDD during the 7-day practice quit attempt.

### Trial design {8}

The current study is a randomized, double-blind, placebo-controlled, 3-arm, parallel-group study. Individuals will be randomly assigned to receive either naltrexone (50 mg QD), varenicline (1 mg BID), or matched placebo for 2 weeks.

## Methods: participants, interventions, and outcomes

### Study setting {9}

This is a single-site trial. All data will be collected within a laboratory setting on the University of California, Los Angeles campus. For relevant biomedical markers (i.e., blood, blood pressure, etc.), collection will take place at the UCLA Clinical and Translation Research Center.

### Eligibility criteria {10}

The inclusion criteria are:

1. Participants must be between the ages of 21 and 65
2. Participants meet diagnostic criteria (four or more symptoms within past 12 months) for moderate or severe alcohol use disorder (AUD) diagnosis according to the current Diagnostic and Statistical Manual of Mental Disorders (DSM-5)
3. Self-report intrinsic motivation to reduce or quit drinking within the next 6 months
4. Report drinking at least 28 drinks per week, if male, or 14 drinks per week, if female, in the 28 days prior to initial consent
5. Participants must have reliable internet access

The exclusion criteria are:

1. Current DSM-5 substance use disorder (SUD) diagnosis for any psychoactive substances other than alcohol and nicotine
2. Lifetime DSM-5 diagnosis of schizophrenia, bipolar disorder or any other psychotic disorder
3. Positive urine screen for any drugs other than cannabis
4. Present clinically significant alcohol withdrawal symptoms at screening visits (indicated by a score of ≥ 10 on the Clinical Withdrawal Assessment for Alcohol-Revised (CIWA-Ar))
5. Participants have an intense fear of needles or any adverse reactions to needle puncture
6. If the participant identifies as female, they must not be pregnant, nursing, or planning to get pregnant while taking part in the study, and must also agree to one of the following methods of birth control (except for individuals who are surgically sterile or post-menopausal):
  - Oral contraceptives
  - Contraceptive sponge
  - Patch
  - Double barrier
  - Intrauterine contraceptive device
  - Etonogestrel implant
  - Medroxyprogesterone acetate contraceptive injection
  - Complete abstinence from sexual intercourse
  - Hormonal vaginal contraceptive ring
7. Present any medical condition (i.e., unstable cardiac, renal, or liver disease, uncontrolled hypertension or diabetes) that may interfere with safe study participation
8. Currently taking any psychotropic medications that may compromise participant safety (determined by the investigators)
9. Currently or have previously taken naltrexone and/or varenicline
10. Present any other circumstances that may compromise participant safety (determined by the investigators)

### Who will take informed consent? {26a}

Consent will be taken by researchers and study physicians trained in Good Clinical Practices (GCP) and according to Health Insurance Portability and Accountability Act Guidelines. Consent to participate in qualitative interviews at the baseline screening visit will be taken by trained research staff. Contingent on eligibility, medical consent will be obtained by study physicians.

### Additional consent provisions for collection and use of participant data and biological specimens {26b}

Consent includes the option to give permission to collect blood for a comprehensive metabolic panel (CMP) and complete blood cell count (CBC) to determine health eligibility. Permission will also be asked for urine collection for toxicology screens and pregnancy tests (if applicable) throughout the length of the study. All biological specimens are collected strictly for eligibility purposes and to ensure protocol adherence. All samples will be disposed immediately after testing.

## Interventions

### Explanation for the choice of comparators {6b}

The purpose of the current study is to develop and validate this novel model to screen novel compounds and advance medications development. Naltrexone (NTX) was chosen to evaluate the novel practice quit attempt model as it is one of the few FDA-approved medications AUD. RCT studies with oral NTX have shown that it reduced drinks per drinking day, alcohol craving, rates of relapse, and the subjective pleasurable effects of alcohol [20–26]. As such, NTX represents a well-known, well-studied medication that is ideal for testing a novel paradigm.

Varenicline (VAR) is a promising pharmacotherapy for the treatment of AUD. VAR has been shown to reduce alcohol self-administration, consumption, and craving [31–33]. A recent RCT of VAR in individuals with AUD found that it reduced drinks per drinking day, alcohol craving, and percentage of heavy drinking days [34]. These studies suggest VAR as a potential AUD pharmacotherapy. The addition of VAR as a third arm in the current study will allow us to further validate this novel practice quit attempt model.

Additionally, the inclusion of a promising pharmacotherapy allows us to compare the efficacy of two medications head-to-head in a cost-effective manner. The 3-arm design of a novel medication (VAR), standard treatment (NTX), and placebo allows us to not only establish efficacy of each medication against placebo, but also of the novel medication again the standard treatment [36]. This study design essentially combines the advantages of placebo and active control studies [37].

### Intervention description {11a}

Participants who are eligible after the physical exam will be randomized to one of three treatment conditions (naltrexone 50 mg, varenicline 2 mg, or placebo). Urn randomization will be stratified by gender, smoking status (as indicated by participant response to question 1 of the Fagerstrom Test for Nicotine Dependence), and drinking status (“heavy” drinker defined as 28 or more drinks per week for males/14 or more drinks per week for females, or “very heavy” drinker, defined as 35 or more drinks per week for males/28 or more drinks per week for females). The UCLA Research Pharmacy will manage the blind. The three treatment conditions will not be different in appearance or method of administration. All participants will undergo a week-long medication titration period prior to the onset of the practice quit attempt as follows: for the naltrexone condition, 12.5 mg will be taken for the first 3 days, followed by 25 mg dosage from days 4–7. The target dosage of 50 mg will be ingested days 8–14. As for the varenicline condition, a dosage of 0.5 mg will be taken for the first 3 days followed by an increase to 1 mg for days 8–14. The intended dosage of 2 mg will be taken days 8–14. Each condition will the instructed to take prescribed medication twice per day as detailed in Table 1.

**Table 1:** Dosing schedule for study medications

Group:	NTX		VAR		PLA	
Day:	AM	PM	AM	PM	AM	PM
**Medication titration**						
**1**	1 capsule (12.5 mg NTX)	1 capsule (placebo)	1 capsule (0.5 mg VAR)	1 capsule (placebo)	1 capsule (placebo)	1 capsule (placebo)
**2**	1 capsule (12.5 mg NTX)	1 capsule (placebo)	1 capsule (0.5 mg VAR)	1 capsule (placebo)	1 capsule (placebo)	1 capsule (placebo)
**3**	1 capsule (12.5 mg NTX)	1 capsule (placebo)	1 capsule (0.5 mg VAR)	1 capsule (placebo)	1 capsule (placebo)	1 capsule (placebo)
**4**	1 capsule (25 mg NTX)	1 capsule (placebo)	1 capsule (0.5 mg VAR)	1 capsule (0.5 mg VAR)	1 capsule (placebo)	1 capsule (placebo)
**5**	1 capsule (25 mg NTX)	1 capsule (placebo)	1 capsule (0.5 mg VAR)	1 capsule (0.5 mg VAR)	1 capsule (placebo)	1 capsule (placebo)
**6**	1 capsule (25 mg NTX)	1 capsule (placebo)	1 capsule (0.5 mg VAR)	1 capsule (0.5 mg VAR)	1 capsule (placebo)	1 capsule (placebo)
**7**	1 capsule (25 mg NTX)	1 capsule (placebo)	1 capsule (0.5 mg VAR)	1 capsule (0.5 mg VAR)	1 capsule (placebo)	1 capsule (placebo)
**Practice quit attempt**						
**8**	1 capsule (50 mg NTX)	1 capsule (placebo)	1 capsule (1 mg VAR)	1 capsule (1 mg VAR)	1 capsule (placebo)	1 capsule (placebo)
**9**	1 capsule (50 mg NTX)	1 capsule (placebo)	1 capsule (1 mg VAR)	1 capsule (1 mg VAR)	1 capsule (placebo)	1 capsule (placebo)
**10**	1 capsule (50 mg NTX)	1 capsule (placebo)	1 capsule (1 mg VAR)	1 capsule (1 mg VAR)	1 capsule (placebo)	1 capsule (placebo)
**11**	1 capsule (50 mg NTX)	1 capsule (placebo)	1 capsule (1 mg VAR)	1 capsule (1 mg VAR)	1 capsule (placebo)	1 capsule (placebo)
**12**	1 capsule (50 mg NTX)	1 capsule (placebo)	1 capsule (1 mg VAR)	1 capsule (1 mg VAR)	1 capsule (placebo)	1 capsule (placebo)
**13**	1 capsule (50 mg NTX)	1 capsule (placebo)	1 capsule (1 mg VAR)	1 capsule (1 mg VAR)	1 capsule (placebo)	1 capsule (placebo)
**14**	1 capsule (50 mg NTX)	–	1 capsule (1 mg VAR)	–	1 capsule (placebo)	–

On study day 1, participants will report to the laboratory to complete the alcohol CR paradigm and receive their first medication dose under direct observation of study staff. They will receive a 7-day supply of study medication in blister packs with AM and PM dosing clearly distinguished. After reaching the target medication dose at the end of 1 week, participants will come to the laboratory on study day 8 to receive their second, 7-day supply of study medication and to begin the 7-day practice quit attempt. Participants will be asked to take the AM dose of study medication on study day 8 in the lab under direct observation of study staff. During the practice quit attempt, participants will complete daily online and phone visits to report on their drinking, mood, and craving for alcohol during the previous day in a daily diary assessment (DDA). For each virtual visit, participants will be contacted over the phone by research staff. Participants will first be asked about adverse events (open-ended) and about use of concomitant medications. Research staff will then administer the CIWA-Ar to measure alcohol withdrawal. Next, they will ask participants to report on their past day drinking as well as cigarette and marijuana use. Finally, while participants are still on the phone, research staff will send a link to the DDA (administered via Qualtrics).

All participants will meet with a trained study counselor briefly after the second cue exposure session on day 14. This brief intervention draws from motivational interviewing and Screening, Brief Intervention, and Referral to Treatment (SBIRT) models. It uses the therapeutic stance of motivational interviewing which is collaborative and client-centered. Consistent with the literature on brief intervention, the therapist will seek opportunities to engage in and amplify change talk. Together, the combination of evidence-based practices and principles applied to AUD, coupled with the experience of change in the context of study participation, is expected to result in an opportunity for health behavior change (i.e., reductions in alcohol use).

### Criteria for discontinuing or modifying allocated interventions {11b}

Criteria for discontinuing or modifying allocated interventions are at the discretion of the study physicians or principal investigator. One week after beginning the medication, physicians will speak with the participant via phone call to check for any adverse events. If reported, participant may either undergo a dose-reduction or termination. Participants will also have the option to voluntarily discontinue all medication at any point. All severe adverse events (SAEs) will be reported to relevant reporting entities immediately.

### Strategies to improve adherence to interventions {11c}

Adherence to interventions is facilitated by dividing the medication into separate blister packs for two distributions, the daily virtual visits during the practice quit attempt period, and a completion bonus. The separation of the study medication into two blister packs, each a 7-day supply, will motivate participants to come back to the laboratory for the second supply, and reduce the chance of them misplacing the medication at the start of the study. During the practice quit attempt period, the participants will be asked to send pictures of their blister packs to the study staff after completion of the daily phone visits. This will allow the study staff to count the medication for compliance. Additionally, a completion bonus will be given out to participants on the last day of the study (day 14) if they have completed at 7 out of the 8 in-person and virtual visits. This is to motivate participants to complete all daily phone visits and online assessments (DDA).

### Relevant concomitant care permitted or prohibited during the trial {11d}

Relevant concomitant care, if it does not interfere with safe study participation, will be permitted during the trial and recorded on a source document.

### Provisions for post-trial care {30}

Post-trial care may continue after study participation if any adverse events remain. The study physician will contact the participant via phone to follow-up and close out any adverse events. Participants who drop out of the trial due to adverse events do not fall into this category.

### Outcomes {12}

The primary outcomes are (1) PDA and/or lower number of DPDD during 7-day practice quit attempt in the NTX (50 mg) or VAR (2 mg) groups, as compared to placebo, (2) reduction in cue-induced craving for alcohol in the NTX (50 mg) or VAR (2 mg) groups, as compared to placebo, and (3) the association between medication effects on CR in the laboratory and drinking behavior during the practice quit attempt. PDA and DPDD are captured via self-reported Timeline Followback from baseline until the close of their practice quit attempt*.* CR will be captured via a cue-exposure paradigm at study day 1 prior to ingesting the first dose of study medication, and on study day 14, approximately 90 min after study drug administration.

Secondary outcomes are PDA and number of DPDD during the 7-day practice quit attempt, as directly compared between NTX (50 mg) and VAR (2 mg).

### Participant timeline {13}

**Fig. 1:**
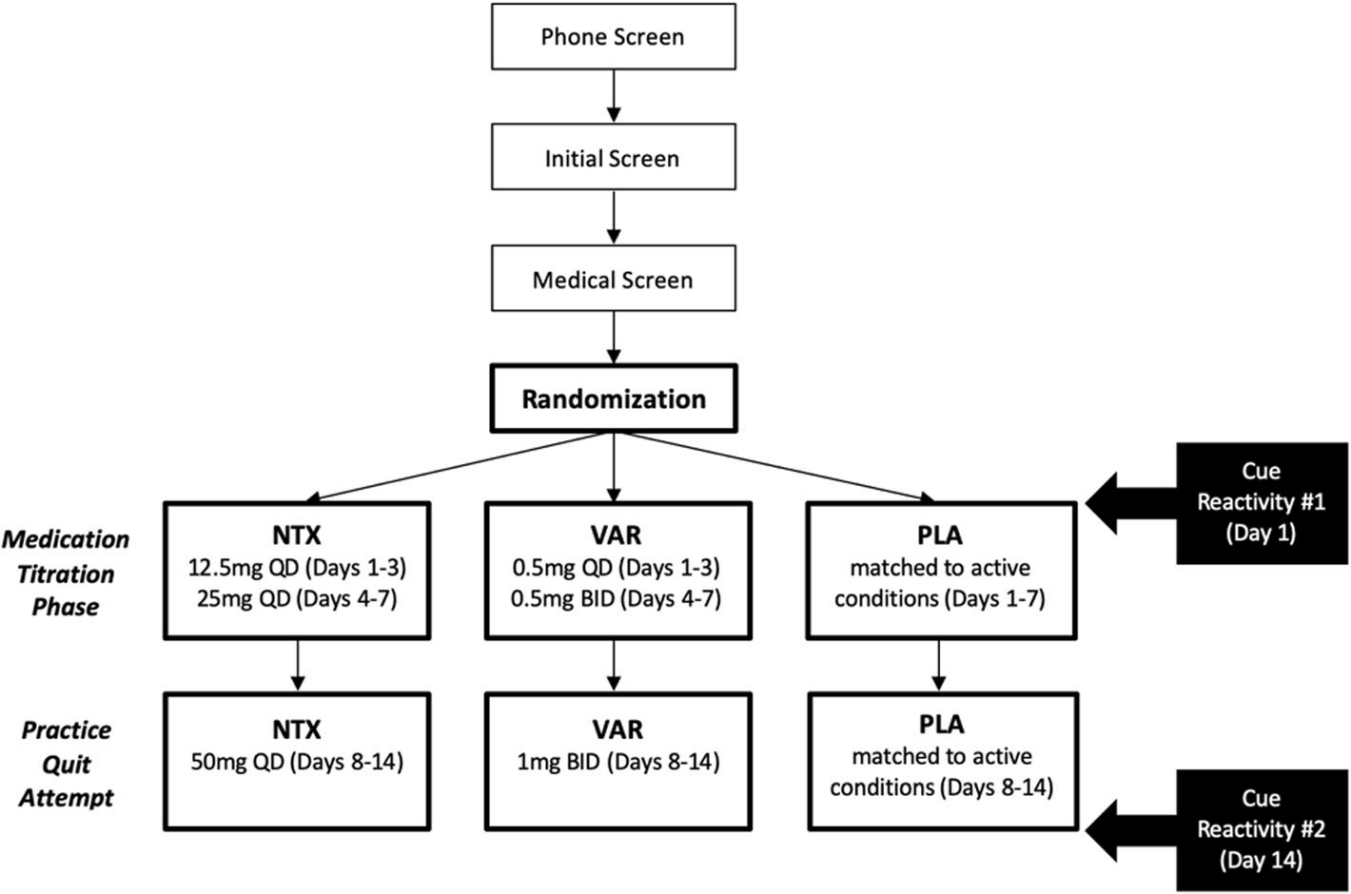
Flow diagram illustrating participant’s timeline through the trial

The study flowchart is presented in Fig. 1. The medication titration phase will take place during the first week of study medication. All eligible participants will come into the laboratory on study day 1 to receive the first half of the study medication (titration), medication instructions, and to take the first dose under the observation of a study staff member. See Table 1 for the dosing schedule of the study medication. Participants will come back a week later (study day 8) to receive their second half of the study medication and begin the practice quit attempt. During this week, participants will be asked complete brief visits over the phone and online every day (study days 9–13). On study day 14, participants will come back to the lab for an in-person visit to close out study participation. Participants will also complete two alcohol CR sessions throughout the study—once on day 1 prior to ingesting the first dose of study medication and again on day 14, approximately 90 days after the last dose of study medication. See Table 2 for schedule of assessments.

**Table 2:** Schedule of assessments

Study visit:	Initial screening	Medical screening	Cr sessions (days 1 and 14)	Randomization (day 1)	Practice quit (days 8–14)
**Screening/individual difference measures:**					
Alcohol Dependency Scale (ADS) [39]				x	
Beck Anxiety Inventory (BAI) [40]				x	x*
Beck Depression Inventory (BDI) [41]				x	x*
Cannabis Use Disorder Identification Test (CUDIT) [42]				x	
Demographics	x				
Fagerstrom Test for Nicotine Dependence (FTND) [43]	x				
Family Tree Questionnaire (FTQ) [44]				x	
Graded Chronic Pain Scale [45]				x	
ImBIBe (shortened version of the Drinker Inventory of Consequences) [46]				x	
Inventory of Drinking Situations (IDS) [47]				x	
Locator Form	x				
Monetary Choice Questionnaire (MCQ) [48]				x	
Obsessive Compulsive Drinking Scale (OCDS) [49]				x	x*
Penn Alcohol Craving Scale (PACS) [50]				x	x*
Perceived Stress Scale (PSS) [51]				x	
Pittsburgh Sleep Quality Index (PSQI) [52]				x	x*
Readiness to Change (RTC) Ladder [53]				x	
Self-Report Habit Index (SRHI) Drinking and Smoking [54]				x	
Structured Clinical Interview for DSM-5 Screener and AUD Module [55]	x				
Timeline Follow Back (TLFB) [56]	x	x		x	x
UCLA Reward Relief Habit Drinking Scale (UCLA RRHDS) [57]				x	
UPPS-P Impulsive Behavior Scale [58]				x	
**Safety measures/biomarkers:**					
Adverse events/SAFTEE [59]					x
Alcohol Breathalyzer	x	x	x	x	x
Birth Control Assessment	x				
Clinical Institute Withdrawal Assessment (CIWA-Ar) [60]	x	x		x	x
Columbia Suicide Severity Rating Scale (C-SSRS) [61]	x				
Comprehensive Metabolic Panel/Complete Blood Count		x			
Concomitant Medications	x	x		x	x
Electrocardiogram (EKG)		x			
Medical History/Physical Exam		x			
Urine Drug Screen	x	x		x	
Urine Pregnancy Test	x	x		x	
Vital Signs	x	x		x	x
**Experimental measures:**					
Alcohol Urge Questionnaire (AUQ) [62]			x		
Daily Diary Assessment					x
Profile of Mood States (POMS) [63]			x		

*Measure collected on day 14 only

### Sample size {14}

Power analyses were conducted using G*Power 3.1.9.2. In order to conduct a one-way ANOVA with fixed effects, we estimated a medium-to-large effect size (*f* = 0.40 and an alpha error probability of 0.05). Specifically, with 3 groups a sample size of 90 completers, the study has an actual power of 0.91%. Therefore, we will to randomize 108 individuals (36 in each group) to reach a final sample of 30 completers per group.

### Recruitment {15}

Participants will be recruited from the community through online and newspaper advertisements, as well as campaigns on multiple social media platforms (Instagram, Craigslist, and Facebook). Campaigns in local buses and print publications (e.g., LA Weekly) will also be implemented. Targeted recruitment will also take place through a lab database of previous study participants who agreed to be contacted for future studies.

### Assignment of interventions: allocation

#### Sequence generation {16a}

Randomization will be done in a 1:1:1 ratio, to NTX, VAR, or placebo using a stratified block randomization procedure. Participants will be stratified by gender, smoking status (as reported on question 1 for the FTND), and drinking status (“heavy drinking” defined as 28 or more drinks per week for males or 14 or more per week for females; “very heavy drinking” defined 35 or more drinks per week for males or 28 or more drinks per week for females). The allocation sequence is computer-generated. Study staff will use a blinded stratification table for participant assignment.

#### Concealment mechanism {16b}

All study medication will be prepared by the UCLA Research Pharmacy and will be identically matched in appearance. The medication labels will not reveal the drug identity.

#### Implementation {16c}

The study staff will enroll the participant to the trial using ONCORE, a UCLA web-based clinical trials management system (CTMS). The study staff will generate the allocation sequence by filling out the blinded stratification table. They will assign a scenario and sequence number according to the participant’s gender, smoking status, and drinking status. The scenario and sequence number will be written on the prescription sent out to the UCLA Pharmacy. The UCLA Pharmacy will randomize the participant according to the unblinded stratification table.

## Assignment of interventions: blinding

### Who will be blinded {17a}

The study will be double-blinded such that trial participants and the research team, including staff, PIs, and physicians, will be naive to the identity of the medication that the participant is receiving. All study medication will be prepared by the UCLA Research Pharmacy and will be identically matched in appearance, and the medication labels will not reveal the drug identity.

### Procedure for unblinding if needed {17b}

Following a severe adverse event or emergency, unblinding may be permissible contingent on the approval of the study physician and/or principal investigator (PI). In the event that significant medical problems are encountered, the study blind will be broken and appropriate medical treatment will be provided.

## Data collection and management

### Plans for assessment and collection of outcomes {18a}

Data are collected at the behavioral eligibility screening visit, the randomization visit (baseline, day 1), at each of the daily phone visits during the practice quit attempt period (days 9–13), and at the in-person study visits (days 8 and 14). All staff personnel will be trained on any relevant assessment procedures and inter-reliability will be monitored continuously by the primary investigator. For the drinking outcomes (i.e., PDA and DPDD), data will be collected via participant self-report through the Timeline Followback [56]. The Alcohol Urge Questionnaire (AUQ) will be used in the CR paradigm to measure craving. The AUQ is an 8-item scale in which participants will rate their present experience of alcohol craving on a 7-point Likert scale [62]. The AUQ has demonstrated high test-retest reliability, high internal consistency, and construct validity in human laboratory studies [62, 64, 65].

### Plans to promote participant retention and complete follow-up {18b}

A completion bonus of $100 will be given to study participants who complete at least 7 out of the 8 in-person and virtual visits during the practice quit attempt period. This is to encourage participant motivation to complete the daily phone visits and online assessments. Any participant who drops out of the study prior to the final visit (day 14) will be invited to come back to provide follow-up data and will be compensated accordingly.

### Data management {19}

Self-report measures will be directly completed through an electronic data capture (EDC) electronic case report forms (eCRF) system, Qualtrics. Timeline Followback data will be entered by research staff into excel in order to generate daily drink averages based on standard drink calculations. All other data will be entered by research staff onto SPSS. Data will be held on a secure server at the University of California, Los Angeles. Appropriately qualified personnel designated by the PI will monitor data entry and ensure that missing data are addressed as soon as possible after detection. All Timeline Followback data will be double-checked by research staff to ensure validity. Excel will also be formulated to detect and notify in the case of any abnormal values.

### Confidentiality {27}

To maintain subject confidentiality, all laboratory specimens, eCRFs, reports, and other records will be identified by a subject number only. Research and clinical records will be stored in a locked cabinet. Only research staff and other required regulatory representatives will have access to the records. Subject information will not be released without written permission.

### Plans for collection, laboratory evaluation, and storage of biological specimens for genetic or molecular analysis in this trial/future use {33}

Blood samples to determine participant physical eligibility, measured via complete metabolic panel and complete blood count, will be collected by the UCLA Clinical Translation and Research Center and will be processed by the UCLA Pathology Research Portal. Storage is not applicable.

## Statistical methods

### Statistical methods for primary and secondary outcomes {20a}

To test the first primary outcome, that pharmacotherapy will improve drinking outcomes, we will conduct a repeated measures ANCOVA on PDA and DPDD predicted by Medication condition (NTX vs. PLAC and VAR vs. PLAC). The covariates are as follows: (1) gender, (2) smoking status (as reported on question 1 of the FTND), and (3) drinking status (“heavy drinking” defined as 28 or more drinks per week for males or 14 or more per week for females; “very heavy drinking” defined 35 or more drinks per week for males or 28 or more drinks per week for females). To test the second primary outcome, that pharmacotherapy will reduce alcohol craving in comparison to placebo, we will conduct a series of repeated measures ANCOVAS on Alcohol Cue – Water cue change scores on the Alcohol Use Questionnaire (AUQ) as predicted by Medication condition (NTX vs. PLAC and VAR vs. PLAC), using the same covariates as above. To test the third primary outcome, that there is an association between medication effects on cue-reactivity and drinking outcomes, a series of regression analyses will be conducted testing whether medication effects on drinking (indicated by NTX – PLAC and VAR – PLAC change scores) are predicted by medication effects on CR (indicated by NTX – PLAC and VAR – PLAC change scores on the CR outcomes described for aim 2).

To test the secondary outcome, we will conduct direct comparisons between the two medication conditions (NTX and VAR) on PDA and DPDD during the practice quit attempt.

### Interim analyses {21b}

There will be no interim analyses.

### Methods for additional analyses (e.g., subgroup analyses) {20b}

The stratification variables involved in the medication randomization process may moderate outcomes. These are three stratification variables: (1) gender, (2) smoking status (as reported on question 1 of the FTND), and (3) drinking status (“heavy drinking” defined as 28 or more drinks per week for males or 14 or more per week for females; “very heavy drinking” defined 35 or more drinks per week for males or 28 or more drinks per week for females).

### Methods in analysis to handle protocol non-adherence and any statistical methods to handle missing data {20c}

If contact is lost, the participant will be counted as non-compliant and coded as a failed quit attempt (i.e., non-abstinent).

### Plans to give access to the full protocol, participant level-data and statistical code {31c}

All data collected in this project will be shared (after appropriate de-identification) with the scientific community in a timely manner, in accordance with NIH Policy. Specifically, the dataset will be made available to the scientific community upon request and a data application will be required.

## Oversight and monitoring

### Composition of the coordinating center and trial steering committee {5d}

The coordinating center is directed by the primary investigator at the UCLA Addictions Laboratory. The UCLA Addictions Laboratory is the site of enrollment, participation, data collection, data management, and study administration, which will take place under the responsibility of research assistants. The research assistants will report directly and meet weekly with the primary investigator and co-investigators. An additional testing site includes the UCLA-Westwood Clinical and Translational Research Center (CTRC) which is the site for physical exams.

### Composition of the data monitoring committee, its role and reporting structure {21a}

Given that all study medications are FDA-approved and taken by study participants for a short duration, it was decided that a data monitoring committed was not warranted for this study.

### Adverse event reporting and harms {22}

Participants will be given a 24-h telephone number to reach the study physician to discuss side effects, and physician office hours will be available as needed. Adverse events, including signs of sickness, will be collected in an open-ended format and coded using a systematic assessment for treatment emergent events (SAFTEE) format at each study visit (in-person and virtual). Vital signs will be monitored at the beginning of each in-person study visit. Alcohol withdrawal will be monitored at each visit through administration of the CIWA-Ar, and any significant withdrawal, as indicated by a score of 10 or more on the CIWA-Ar, will be reported to the study physician immediately. In the event that significant medical problems are encountered, the study blind will be broken and appropriate medical treatment will be provided.

### Frequency and plans for auditing trial conduct {23}

The PI will designate appropriately qualified personnel to periodically perform quality assurance checks at mutually convenient times during and after the study. These monitoring visits provide the opportunity to evaluate the progress of the study and to obtain information about potential problems. The monitor will assure that data are accurate and in agreement with any paper source documentation used, verify that subjects’ consent for study participation has been properly obtained and documented, confirm that research subjects entered into the study meet inclusion and exclusion criteria, verify that study procedures are being conducted according to the protocol guidelines, monitor review AEs and SAEs, and assure that all essential documentation required by GCP guidelines are appropriately filed. At the end of the study, they will confirm that the site has the appropriate essential documents on file and advise on storage of study records.

### Plans for communicating important protocol amendments to relevant parties (e.g., trial participants, ethical committees) {25}

This study was approved by the UCLA Medical IRB 3. Any amendments to the study protocol will be prepared by study staff in collaboration with the PI and Co-Investigators. Amendments will be submitted for approval to the UCLA IRB.

### Dissemination plans {31a}

We will develop a publication and dissemination plan to include conference presentation(s) and journal publication(s).

## Discussion

Alcohol use disorder is a chronic condition with both high relapse and low treatment rates [1, 3]. Despite the significant public health burden, there are few pharmacological treatment options, with only 4 medications receiving FDA approval [7, 9]. This contrast between public need and lack of approved pharmacological treatments does not highlight a lack of research; on the contrary, close to 2 dozen potential medications have reached clinical testing [5]. It instead is largely owed to the presence of an expensive and burdensome medications development process, notoriously deemed the “valley of death,” whereby medications fail in their transition from preclinical to initial clinical testing [9]. There is a second “valley of death” where medications fail to translate from early human laboratory efficacy into large-scale, ecologically valid clinical trials. Therefore, the practice quit attempt model aims to develop a novel early efficacy paradigm to more efficiently screen future AUD medication candidates.

The present study will utilize naltrexone (NTX), an FDA-approved medication for AUD, to serve as an active control to test both the practice quit attempt paradigm and the efficacy of Varenicline (VAR). In relation to the former, NTX is an FDA-approved opioid antagonist with high affinity for both the mu-opioid and kappa-opioid receptors [17]. With its endogenous opioid blocking effects, NTX has been found to be associated with reduction in both alcohol craving and consumption [66, 67]. These effects make NTX an excellent candidate for the practice quit paradigm. VAR is an FDA-approved medication for smoking cessation that has been associated with reduction in alcohol cravings in previous animal and human laboratory studies [32, 33]. Based on these findings and in combination with past literature, VAR poses a potential benefit as an AUD pharmacological therapy and, subsequently, an appropriate experimental medication within the practice quit paradigm.

Earlier screening models for phase 2 medication trials largely lack the ecological validity needed to construct clinically meaningful endpoints for treatment-seeking individuals. This practice quit study differs from previous trials in its introduction of (1) a paradigm that displays assay sensitivity via placebo controls, (2) a superiority comparison between an FDA-approved medication and an experimental candidate, (3) increased ecological validity as participants are asked to quit drinking in the real-world and not only evaluated in the laboratory setting, similar to what is seen in large scale RCTs, and (4) an alcohol CR assessment to validate the sensitivity of the paradigm for detecting medication effects. The successful completion of this study will advance medications development by proposing and validating a novel early efficacy model for screening AUD pharmacotherapies, which in turn can serve as an efficient strategy for making go/no-go decisions as to whether to proceed with clinical trials. Specifically, a valid model of initial efficacy will allow us to reliably detect an efficacy signal for AUD pharmacotherapies, and in turn decide whether to proceed to the full-scale efficacy (phase 2) testing.

## Trial status

Recruitment started on 21 January 2020 and is currently ongoing. The current protocol is version 5 (dated 05/2020). Project end date is projected to be 31 August 2021.

## Abbreviations

AUD: Alcohol use disorder
FDA: Federal drug administration
NTX: Naltrexone
CR: Cue-reactivity
VAR: Varenicline
PLAC: Placebo
RCT: Randomized controlled trial
UCLA: University of California, Los Angeles
QD: Once daily
BID: Twice daily
DSM-5: Diagnostic and Statistical Manual of Mental Disorders
CIWA-Ar: Clinical Withdrawal Assessment for Alcohol-Revised
SUD: Substance use disorder
CMP: Complete metabolic panel
CBC: Complete blood cell count
BrAC: Breath alcohol
EtG: Ethyl glucuronide
PDA: Percent days abstinent
DPDD: Drinks per drinking day
SBIRT: Screening, Brief Intervention, and Referral to Treatment
SAEs: Severe adverse events
AEs: Adverse events
CTMS: Clinical trials management system
AUQ: Alcohol Urge Questionnaire
ADS: Alcohol Dependency Scale
BAI: Beck Anxiety Inventory
BDI: Beck Depression Inventory
CUDIT: Cannabis Use Disorder Identification Test
FTND: Fagerstrom Test for Nicotine Dependence
FTQ: Family Tree Questionnaire
IDS: Inventory of Drinking Situations
MCQ: Monetary Choice Questionnaire
OCDS: Obsessive Compulsive Drinking Scale
PACS: Penn Alcohol Craving Scale
PSS: Perceived Stress Scale
PSQI: Pittsburgh Sleep Quality Index
RTC: Readiness to Change
SRHI: Self-Report Habit Index
TLFB: Timeline Follow Back
RRHDS: Reward Relief Habit Drinking Scale
C-SSRS: Columbia Suicide Severity Rating Scale
EKG: Electrocardiogram
POMS: Profile of Mood States
eCRFs: Electronic case report forms
NIH: National Institute of Health
CTRC: Clinical and Translational Research Center
SAFTEE: Systematic Assessment for Treatment Emergent Events
GCP: Good Clinical Practices
IRB: Institutional Review Board
